# Absolute blood levels and kinetics of neurofilament light (NFL) chains for neurological prognosis in comatose patients after cardiac arrest

**DOI:** 10.1186/s13613-025-01491-7

**Published:** 2025-05-30

**Authors:** Timothée Ayasse, Maxime Touron, Marie-Céline Blanc, Estelle Pruvost-Robieux, Jean-Baptiste Lascarrou, Clara Vigneron, Jean-Paul Mira, Frédéric Pène, Alain Cariou, Sarah Benghanem

**Affiliations:** 1 Medical Intensive Care Unit, Cochin Hospital, AP-HP Centre, 27 rue du Faubourg Saint Jacques, Paris, 75014 France; 2 Clinical Chemistry Department, Cochin Hospital, AP-HP Centre, Paris, France; 3 Neurophysiology and Epileptology Department, GHU Paris – Sainte Anne, Paris, France; 4 Université Paris Cité, Paris, France; 5 INSERM 1266 – Institute of Psychiatry and Neurosciences of Paris (IPNP), Paris, France; 6 Medical Intensive Care Unit, University Hospital Center, Nantes, France; 7 INSERM U970 (team 4), Paris Cardiovascular Research Center, Paris, France; 8 AfterROSC Network, Paris, France

**Keywords:** Cardiac arrest, Coma, Neuropronostication, Neurofilament light chain, Neuron-specific enolase, Biomarkers

## Abstract

**Introduction:**

Despite current guidelines, the prognosis of comatose patients after cardiac arrest (CA) remains indeterminate in approximately 50% of patients. Neurofilament light (NFL) chain, a biomarker of neuronal injury, appears to be a promising tool with better prognostic value compared to neuron-specific enolase (NSE). However, further studies are required. The objective was to evaluate the prognostic value of absolute blood levels and kinetics of NFL measured at 24, 48 and 72 h after CA to predict outcome.

**Methods:**

A prospective study conducted at a tertiary CA center conducted between April 2023 and November 2024, including 67 comatose patients after CA with at least one blood NFL measurement. The primary outcome was neurological outcome according to the “best” modified Rankin scale (mRS) within 3 months, with a mRS 0–3 defining a good outcome.

**Results:**

Participants were 64 years old (IQR [53–75]), and 67% were male. 60% of them had out-of-hospital CA and 42% had an initial shockable rhythm. NFL levels were significantly higher in patients with unfavorable outcome compared to those with favorable outcome at each time point (24 h: 256.0 [96.2–441.9] versus 37.9 [17.4–104.5] pg/mL, *p* < 0.001; 48 h: 1297.7 [137.6–3605.0] versus 36.4 [19.3–174.0] pg/mL, *p* < 0.001; 72 h: 1591.9 [350.6–4913.5] versus 49.3 [23.2–146.4] pg/mL, *p* < 0.001). NFL levels at 24 and 48 h showed moderate prognostic accuracy (AUC 0.80 and 0.88, respectively), while NFL at 72 h demonstrated high accuracy (AUC 0.90). For predicting poor outcome, NFL level at 24 h > 250 pg/mL had a specificity of 0.90 [0.75–0.90] and a sensitivity of 0.51 [0.35–0.67]. At 48 h, NFL > 383 pg/mL had a specificity of 0.94 [0.81–1.00] and a sensitivity of 0.66 [0.50–0.81]. At 72 h, NFL > 510 pg/mL presented a specificity of 1.00 [1.00–1.00] and a sensitivity of 0.74 [0.52–0.91]. To predict a good outcome, NFL levels < 82 pg/mL at 24 h, < 307 pg/mL at 48 h and < 459 pg/mL at 72 h after CA were the best compromise between specificity and sensitivity. NFL kinetic was not associated with neurological outcomes (AUC 0.50), whereas NSE kinetic measured between 24 and 72 h showed high prognostic accuracy (AUC 0.92).

**Conclusion:**

In comatose patients after CA, NFL levels at 24, 48 and 72 h could predict both unfavorable and favorable outcomes. Conversely, only the kinetic of NSE levels between 24 and 72 h after CA was associated with neurological outcome.

**Supplementary Information:**

The online version contains supplementary material available at 10.1186/s13613-025-01491-7.

## Introduction

Cardiac arrest (CA) remains a global public health concern, with survival rates at hospital discharge not exceeding 10 to 20% in European countries [1]. Hypoxic-ischemic brain injury (HIBI) is the leading cause of death among patients resuscitated from CA and is associated with long-term disability in survivors [2, 3]. Accurate prognostication of neurologic outcome is crucial to avoid both inappropriate withdrawal of life-sustaining treatment (WLST) in patients who might achieve a favorable outcome and disproportionate care in patients with severe and irreversible HIBI. In case of persistent coma lasting 72 h or more after the return of spontaneous circulation (ROSC), current European guidelines recommend a multimodal neuroprognostication approach to predict poor outcome [4]. This approach involves the use of several markers that include clinical examinations (status myoclonus ≤ 72 h and bilateral absence of pupillary and corneal reflexes ≥ 72 h), bilateral absence of N20 on somatosensory evoked potentials (SSEPs), presence of highly malignant electroencephalogram (EEG) > 24 h, diffuse and extensive anoxic injury on brain imaging, and blood level of neuron-specific enolase (NSE) > 60 µg/L at 48 and/or 72 h [4]. Despite the use of this multimodal algorithm, the prognostic remains indeterminate in 31 to 68% of patients [5–7]. It therefore seems essential to improve neuroprognostication capabilities in this population. To reduce prognostic uncertainly, it would also be necessary to have markers that allow to predict a favorable outcome [8].

Regarding biomarkers, NSE is currently the only one recommended, a NSE > 60 µg/L at 48 and/or 72 h being highly predictive of an unfavorable outcome. Nevertheless, this threshold varies from 33 to 120 µg/L in studies, permitting a high specificity despite a limited sensitivity [9]. Moreover, the use of NSE has limitations because some situations (notably hemolysis and neuroendocrine tumors) can lead to falsely high levels [10]. Other biomarkers are now available, such as neurofilament light chain (NFL), a neuron-specific axonal protein, that has recently been assessed as a predictor of neurological outcome after CA [10–13]. This biomarker has shown higher prognostic value at 48 and/or 72 h compared to NSE and other biomarkers of glial injury, such as glial fibrillary acidic protein (GFAP) and S100 beta protein (pS100b) [10, 14]. Moreover, early NFL levels (collected at 12 and/or 24 h after ROSC) may be highly predictive of neurological outcome, unlike early NSE [15].

Most of these studies are based on ancillary studies of randomized controlled trials, which over-represent patients with favorable prognostic factors such as shockable rhythm and CA due to acute coronary syndrome (ACS). Therefore, further studies are required before this biomarker can be used routinely. Finally, although the kinetics of NSE during the first 72 h post-ROSC appear to be associated with both favorable (when decreasing) and unfavorable outcomes (when increasing) [16], kinetics of NFL and its prognostic value have never been evaluated.

In the present study, we aimed to evaluate the prognostic value of NFL measured at 24, 48 and 72 h after CA for predicting unfavorable and favorable neurological outcome. We also aimed to assess the prognostic value of ascending and descending trends of NFL over time for predicting unfavorable and favorable outcomes, respectively. Finally, we compared the prognostic accuracy of NFL and NSE.

## Methods

### Study design and patients

This was a prospective study conducted in a tertiary cardiac arrest center (Cochin Hospital, AP-HP Centre, Paris, France). We included all patients who were comatose (i.e., GCS < 8) at ICU admission after an in-hospital (IHCA) or out-of-hospital (OHCA) cardiac arrest between April 2023 and November 2024 and who had at least one serum NFL level measurement at 24, 48 and/or 72 h after ROSC. Exclusion criteria were age < 18 years and established brain death.

### Data collection

This observational study followed the STROBE guidelines [17]. Demographic and clinical data were collected from medical records and local databases. CA management data were collected using Utstein style, including characteristics of the CA (location, bystander witnessed), initial cardiac rhythm and time to ROSC. The in-hospital variables included causes of CA, clinical characteristics on ICU admission (SAPS II, arterial pH and arterial lactate level), ICU mortality, cause of death and decisions to withdraw life-sustaining therapies (WLST). Patients’ relatives were informed that the data were collected for clinical research purposes. The research protocol was approved by the ethics committees (2019-A01378-49, CPP-SMIV-190901; 2022-A01811 42; CPP-Ile-de-France-I) and the French data protection authorities, in accordance with the Declaration of Helsinki.

### Patients’ management

Patient management was standardized and followed the 2021 ERC-ESICM guidelines [4]. Targeted temperature management (TTM) at 36 °C was initiated as soon as possible after admission and maintained for 24 h using an external cooling device. The sedation protocol involved short-acting drugs (propofol and remifentanil), with adjustments based on the Richmond Agitation-Sedation Scale (RASS) [18]. Sedation was discontinued when the patient’s spontaneous body temperature was restored. Patients’ management also included the prevention of secondary cerebral injury from systemic causes (ESM1). Awakening was defined as three consecutive RASS scores of -2 or higher and a reproducible response to the verbal command.

### Neuroprognostication procedure

In the case of persistent coma at 72 h after CA not explained by confounding factors, a multimodal prognostication procedure based on the 2021 ERC-ESICM guidelines [4] was initiated. An unfavorable neurological outcome was considered when at least two of the following conditions were observed: no pupillary and corneal reflexes ≥ 72 h, bilaterally absent N20 on SSEP, highly malignant EEG > 24 h, status myoclonus ≤ 72 h, diffuse and extensive anoxic injury on brain imaging, NSE > 60 µg/L at 48 and/or 72 h [5] (ESM2 and Figure S1).

### Biomarkers assessment

NFL and NSE were measured in serum samples collected at 24 h, 48 h and/or 72 h after admission. After collection in gel separator tubes, whole blood samples were centrifuged within 4 h. Serum was stored at + 4–8 °C for analysis within 6 h. In case of delayed analysis (within 48–72 h because of the weekend, for example), serum samples were stored at -40 °C. Whole blood samples were collected and centrifuged at 2000 g, 4 °C, 10 min and separated sera were obtained. The delay between collection and centrifugation was no longer than 3 h. Assay was performed either immediately after centrifugation (NSE) or after storage at 4 °C for no longer than 3 days (NFL).

The NSE immunoassay was performed on Cobas^®^ analyzer using the sandwich immunochemiluminescent Elecsys NSE assay (Roche Diagnostics, Meylan, France). The measuring range of NSE assay is 0.22–300 µg/L. The 95th percentile value is 16.3 µg/L, which is considered normal (data from manufacturer). In our laboratory, within-run and between‐run imprecision were < 1.0% and < 6.0% respectively, for concentrations of about 12 and 100 µg/L. Hemolyzed samples were not included due to overestimation of NSE. The laboratory is a participating member of the ProBioQual external quality program.

The NFL immunoassay was performed on Lumipulse G600 analyzer using the sandwich immunoenzyme chemiluminescent NFL assay (Fujirebio, ZA Courtaboeuf, France). The measuring range of NFL assay is 4-5000 pg/mL. Within-run and between‐run imprecision were < 5% and < 7.0% respectively, for concentrations of about 18 and 800 pg/mL. The laboratory is a participating member of the Alzheimer’s Association QC program for CSF and blood biomarkers. In our clinical practice, NFL is routinely measured as an exploratory biomarker for neuronal injury. Consequently, physicians are not blinded to NFL values. However, NFL is not included in the neuroprognostication algorithm due to the absence of established guidelines defining its role in the multimodal clinical decision-making process following CA. Given the limited published data on serum biomarker kinetics, we have taken a pragmatic approach in our analysis that is suitable for daily clinical practice. Therefore, we assessed biomarker kinetics using either two or three consecutive samples. For NSE kinetics, the trend was classified as “descending” if the two or three successive samples showed a decreasing pattern, and “ascending” if the samples showed an increasing pattern. For NFL kinetics, given that animal studies suggest a peak at 48 h [19], we defined an “ascending trend” as an absolute increase in NFL levels between 24 and 48 h, and a “descending trend” as an absolute decrease between 24 and 48 h. Biomarkers kinetics were considered as not assessable when the trend was discordant across three consecutive samples.

### Other prognostic markers assessment

Clinical neurological examination was performed several times a day and included the assessment of pupillary and corneal reflexes. Intermittent EEG was recorded in the ICU by an experienced technician using a Micromed Brain Quick device (Micromed^®^ SAS, Treviso, Italy) with 19 electrodes, for 20 min. Standardized terminology from the 2021 American Clinical Neurophysiology Society (ACNS) was used to describe the background activity, reactivity, and the presence of superimposed additional features such as rhythmic or periodic patterns, seizures and status epilepticus (SE) [20]. Each recording was then classified according to Westhall et al. into one of the mutually exclusive categories, namely highly malignant (suppression or burst-suppression background, with or without superimposed periodic discharges), malignant (presence of at least one of the following: abundant and generalized rhythmic or periodic discharges, seizures or SE, discontinuous or low-voltage background, absence of reactivity and inversion of the anteroposterior gradient) or benign (absence of malignant and highly malignant features) [21]. SSEPs were recorded in the ICU by an experienced technician. The median nerve was electrically stimulated using a Micromed Brain Quick device (Micromed^®^ SAS, Treviso, Italy) and ascending signals were recorded from the peripheral plexus brachialis, cervical level, subcortical level and the somatosensory cortex (N20 response). All EEG and SSEP recordings were analyzed by experienced neurophysiologists, blinded to the other prognosis markers.

### Outcomes

The primary outcome was neurological outcome according to the “best” modified Rankin scale (mRS) within 3 months. Briefly, the mRS is a 7-point scale of global disability ranging from 0 (no symptoms) to 6 (death). An mRS level between 0 (no symptom) and 3 (moderate disability) was considered as a favorable outcome. We used the “best” mRS observed within 3 months; in fact, death after awakening can be observed in this critically-ill population, these patients being misclassified as mRS 6 (i.e., poor neurological outcome) [22]. The mRS was assessed by an independent trained researcher by telephone interview with the patient or their next of kin. If the information could not be obtained by telephone interview, we collected the neurologic result from the medical records.

### Statistical analysis

Results are expressed as numbers (percentages) for categorical variables and as medians (and interquartile ranges) for continuous variables. All data were tested for normal distribution using the Shapiro-Wilk test. Usual parameters of multimodal neuroprognostication were compared between the two groups using the Wilcoxon signed-rank test. To explore potential selection bias, we compared (1) included and excluded patients; (2) patients who died following WLST related to a presumed severe HIBI and other patients.

The prognostic accuracy of blood-based biomarker levels and other components of the prognostication algorithm for predicting either unfavorable or favorable neurological outcome was assessed using receiver operating characteristic (ROC) curves. The area under the curve (AUC), specificity (Sp), sensitivity (Se), false-positive (FP), true-positive (TP), false-negative (FN), true-negative (TN) values were calculated for each ROC curve. Positive predictive value (PPV), negative predictive value (NPV), false positive rate (FPR), and false negative rate (FNR) were also calculated. High accuracy was defined as an AUC > 0.90, moderate accuracy as an 0.70 > AUC > 0.90 and low accuracy as an 0.50 > AUC > 0.70 [23]. Because high specificity (and low FPR) is important for poor outcome prediction, we identified two thresholds: one that ensures a 100% specificity, and another that balances optimized sensitivity with a specificity greater than 90%, called “optimal specificity”. We also determined the best thresholds using the Youden index and analyzed the NSE level > 60 µg/L, according to guidelines [4]. Because high sensitivity is important for good outcome prediction, we identified two thresholds: one that ensures a 100% sensitivity, and another that balances optimized sensitivity and specificity using the Youden index; we also analyzed two predefined thresholds: NSE < 17 µg/L at 24, 48 and 72 h [24] and NSE < 41 µg/L at 48 h [25].

We compared the prognostic accuracy of NFL and NSE values for predicting unfavorable and favorable outcome by comparing their AUC values with DeLong’s test. We described the biomarkers levels over time in patients with good versus poor outcomes and illustrated the relationship and discrepancies between NFL and NSE across all time points using a scatter plot.

To assess the added value of NFL and NSE levels for prognosis compared to clinical prediction models, three models were constructed: (1) a clinical model (the Cardiac Arrest Hospital Prognosis - CAHP score) including Utstein variables known to be associated with prognosis (age, out- or in-hospital CA, shock able rhythm, witnessed CA, no-flow and low-flow times, and total dose of adrenaline received during cardiopulmonary resuscitation) [26]; (2) Second, a model with median NFL or NSE levels at 48 h; (3) Third, a model combining CAHP score and biomarkers. The added value of the biomarkers over the clinical prediction score was assessed by comparing AUC.

All statistical analyses were performed with R statistical software version 4.4.0 (R Core Team, 2024, R Foundation for Statistical Computing, Vienna, Austria, https://www.R-project.org). The construction of ROC curves and associated statistics was performed with the R package ‘pROC’, version 1.18.0. A p-value less than 0.05 was considered significant.

## Results

### Study population

During the study period, a total of 173 patients were admitted in the ICU in a comatose state after CA and were screened for participation. Brain death was diagnosed in 9 patients and NFL was not measured in 97 patients (due to availability and feasibility reasons), resulting in 67 patients in the final analysis (ESM3, Figure S2). Compared with included patients, excluded patients presented more frequently with an initial rhythm of pulseless electrical activity (5% vs. 23%, *p* < 0.001) and had a better neurological outcome (49% vs. 25%, respectively, *p* < 0.001) (ESM3, Table S1). Excluded patients due to brain death had high median levels of NFL (235.0, 849.6 and 4572.0 pg/mL) and NSE (107, 281.5, 300.0 µg/L) at 24, 48 and 72 h respectively (ESM3, Table S2).

Among the 67 patients included, 17 patients had one NFL measurement, 19 had two, and 31 had three. Among patients with at least one NFL measurement, 3 had no NSE measurement, 15 had one, 17 had two, and 32 had three. The clinical characteristics of the patients are presented in Table 1. A non-shockable rhythm was observed in 38 patients (58%). The median time to ROSC was 21 [10–34] minutes. The ICU length of stay was 4 (3–7) days. During ICU stay, 40 patients (60%) died, including 25 patients (64%) after a decision of WLST. The main reason for WLST was a presumed severe HIBI (72%). At 3 months, 51 patients (76%) had an unfavorable neurological outcome. Comparison of demographic characteristics and biomarkers levels between patients who died after WLST related to a presumed severe HIBI and other patients highlighted that median time to ROSC, use of epinephrine, median NFL and NSE levels at 48 and 72 h were significantly different between groups (ESM3 - Table S3).

**Table 1:** Patients’ clinical characteristics according to neurological outcome

	All patients n = 67	Favorable outcome n = 16	Unfavorable outcome n = 51	*p* value
Age – yr (IQR)	64 [53–75]	61 [51–76]	66 [56–74]	0.341
Sex – no. (%)				0.878
Men	46 (68.6)	14 (87.5)	31 (60.8)	
**Location at cardiac arrest** Missing data n = 1				**0.022**
Place of residence	22 (32.8)	2 (12.5)	20 (40)	
Public place	18 (26.9)	7 (43.7)	11 (22)	
Hospital	16 (23.9)	2 (12.5)	14 (28)	
Other	10 (14.9)	5 (31.2)	5 (10)	
Bystander-witnessed cardiac arrest	62 (92.5)	16 (100)	46 (90)	0.362
Bystander-performed CPR	54 (80.6)	15 (93.7)	39 (78)	0.310
**First monitored rhythm – no. (%)**				**0.010**
Shockable rhythm	28 (41.8)	15 (93.7)	13 (25.5)	
Non shockable rhythm	38 (56.7)	7 (43.7)	31 (60.8)	
Pulseless electrical activity	2 (3)	0 (0)	2 (6.4)	
Asystole	36 (53.7)	7 (100)	29 (93.5)	
**Median time from cardiac arrest to sustained ROSC (IQR) – min**	**21 [10–34]**	**19 [8–21]**	**29 [20–39]**	**0.004**
No flow	0 [0–5]	0 [0–3]	1 [0–5]	0.172
**Low flow**	**19 [9–30]**	**13 [8–20]**	**26 [14–31]**	**0.008**
**Epinephrine – no. (%)**	**52 (77.6)**	**11 (68.7)**	**41 (80.4)**	**< 0.001**
**SAPS II**	**72 [63–84]**	**69 [59–75]**	**77 [65–86]**	**0.048**
**Arterial pH**	**7.23 [7.16–7.34]**	**7.31 [7.26–7.39]**	**7.20 [7.15–7.28]**	**0.006**
**Arterial lactate level – mmol/liter**	**3.7 [1.7–9.6]**	**1.8 [1.4–3.5]**	**8.2 [3.0–13.1]**	**< 0.001**
ICU length of stay (IQR) – days	4 [3–7]	4 [2–9]	4 [3–7]	0.202
**Best mRS at 3 months– no. (%)**				**< 0.001**
0	13 (19.4)	13 (81.2)	0 (0)	
1	–	–	–	
2	1 (1.5)	1 (6.2)	0 (0)	
3	2 (3)	2 (12.5)	0 (0)	
4	11 (16.4)	0 (0)	11 (21.6)	
5	7 (10.4)	0 (0)	7 (13.7)	
6	33 (49.2)	0 (0)	33 (64.7)	
**ICU mortality – no. (%)**	**40 (59.7)**	1 (6.2)*	**39 (76.5)**	**< 0.001**
Cause of death – no. (%)				0.400
Multiple organ dysfunction syndrome	14 (35)	1 (100)	13 (33.3)	
WLST	25** (62.5)	0 (0)	25 (64.1)	
**Reason for WLST – no (%)**	32** (47.7)	0 (0)	32 (62.7)	**< 0.001**
Hypoxic ischemic brain injury	23 (71.8)	0 (0)	23 (71.8)	
Comorbidities	9 (28.1)	0 (0)	9 (28.1)	

Values are median [interquartile range] and number (percentage)

IQR: interquartile range; ROSC: return of spontaneous circulation; SAPS: simplified acute physiology score; ICU: intensive care unit; mRS: modified Rankin Scale; WSLT: withdrawal of life-sustaining treatment. *1 patient with favorable neurological outcome died in the ICU following multiple organ dysfunction syndrome. **All patients with a decision of WLST were not dead during ICU stay

Compared to patients with a favorable outcome, patients with an unfavorable neurological outcome were more likely to presented a non-shockable rhythm (70% versus 32%, *p* = 0.010), a longer median time to ROSC (29 [20–39] minutes versus 19 [8–21] minutes, *p* = 0.004) and a more frequent use of pre-hospital epinephrine (93% versus 50%, *p* < 0.001). At admission, median arterial lactate levels (8.2 [3–13.1] mmol/L vs. 1.8 [1.4–3.5] mmol/L *p* < 0.001), arterial pH (7.20 [7.15–7.28] vs. 7.31 [7.26–7.39], *p* = 0.004) and SAPS II (77 [65–86] vs. 69 [59–75], *p* = 0.048) were different between groups. There were no significant differences in medical history, ICU length of stay, or ICU management, including the use of targeted temperature management and norepinephrine (Table 1).

### NFL and NSE levels according to neurological outcome

**Fig. 1:**
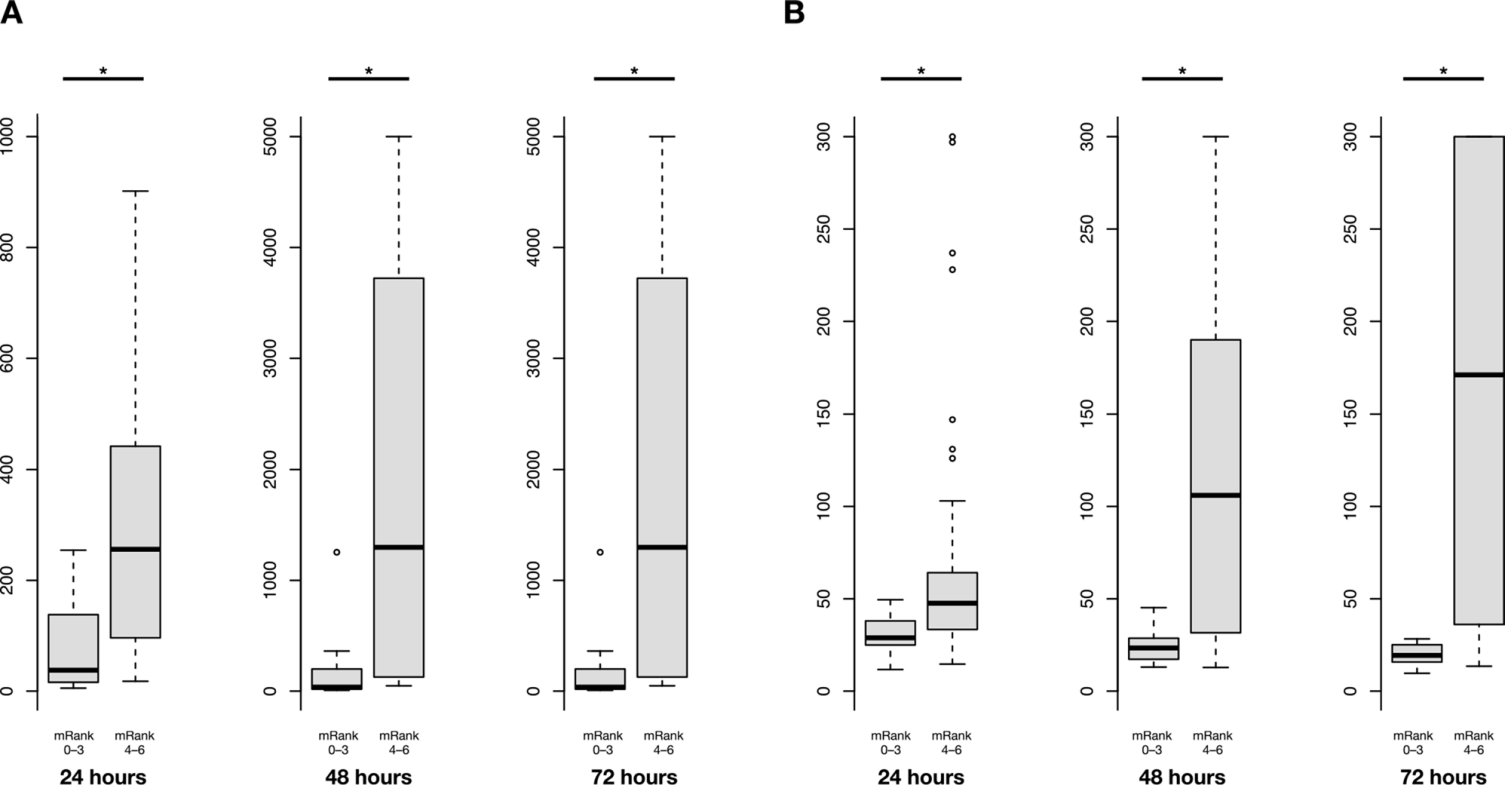
NFL **(A)** and NSE **(B)** levels at 24, 48 and 72 h according to neurological outcome. Favorable neurological outcome: best modified Rankin scale level at 0 to 3 within 3 months; Unfavorable neurological outcome: best modified Rankin scale level at 4 to 6 within 3 months. *: *p* < 0.001. NFL was expressed in pg/mL; NSE was expressed in µg/L

Median serum NFL levels at 24, 48 and 72 h according to neurological outcome are presented in Fig. 1A. NFL levels were higher in patients with unfavorable outcome compared to those with favorable outcome at each time point (24 h: 256.0 [96.2–441.9] versus 37.9 [17.4–104.5] pg/mL, *p* < 0.001; 48 h: 1297.7 [137.6–3605.0] versus 36.4 [19.3–174.0] pg/mL, *p* < 0.001; 72 h: 1591.9 [350.6–4913.5] versus 49.3 [23.2–146.4] pg/mL, *p* < 0.001).

Median serum NSE levels at 24, 48 and 72 h according to neurological outcome are presented in Fig. 1B. NSE levels were higher in patients with unfavorable outcome compared to those with favorable outcome at each time point (24 h: 47.6 [33.4–64.1] versus 28.9 [25.8–37.5] µg/L, *p* < 0.001; 48 h: 106.0 [32.9–185.0] versus 23.4 [17.3–27.4] µg/L, *p* < 0.001; 72 h: 171.0 [36.7–300.0] versus 19.4 [16.3–25.0] µg/L, *p* < 0.001).

Absolute levels and kinetics of NFL and NSE over time according to neurological outcome are presented in ESM3 - Figure S3. The kinetics of NSE showed an increasing trend in cases of unfavorable outcome and a descending trend in cases of favorable outcome, whereas the kinetics of NFL was ascending in both groups.

### Prognostic accuracy of NFL levels for predicting unfavorable outcome

The performance of NFL values at 24, 48, 72 h to predict unfavorable outcome are presented in Table 2:

**Table 2:** Prognostic performance of NFL levels in predicting unfavorable outcome

Time, h	Method	AUC	Cutoff level (pg/mL)	Specificity	Sensitivity	PPV	NPV	Patients, No (%)				
								TP	FN	FP	TN	FPR
**24**	**Spe. at 1**	**0.80 [0.68–0.93]**	**1121**	**1.00 [1.00–1.00]**	**0.13 [0.02–0.63]**	**1.00 [1.00–1.00]**	**0.34 [0.32–0.56]**	**6 (10)**	**37 (59)**	**0 (0)**	**20 (32)**	**0 [0–0]**
	**Optimal Spe**		**250**	**0.90 [0.75–0.90]**	**0.51 [0.35–0.67]**	**0.92 [0.81–1.00]**	**0.46 [0.38–0.56]**	**22 (35)**	**21 (33)**	**2 (3)**	**18 (29)**	**10 [0–25]**
	Youden		82	0.75 [0.60–0.95]	0.81 [0.56–0.93]	0.88 [0.80–0.97]	0.65 [0.48–0.85]	35 (56)	8 (13)	5 (8)	15 (24)	25 [5–40]
**48**	**Spe. at 1**	**0.88 [0.77–0.98]**	**1324**	**1.00 [1.00–1.00]**	**0.56 [0.34–0.78]**	**1.00 [1.00–1.00]**	**0.53 [0.43–0.70]**	**18 (38)**	**14 (29)**	**0 (0)**	**16 (33)**	**0 [0–0]**
	**Optimal Spe**		**383**	**0.94 [0.81–1.00]**	**0.66 [0.50–0.81]**	**0.96 [0.86–1.00]**	**0.58 [0.47–0.71]**	**21 (44)**	**11 (23)**	**1 (2)**	**15 (31)**	**6 [0–19]**
	Youden		307	0.88 [0.63–1.00]	0.78 [0.59–1.00]	0.91 [0.82–1.00]	0.67 [0.52–1.00]	25 (52)	7 (15)	2 (4)	14 (29)	13 [0–38]
**72**	**Spe. at 1**	**0.90 [0.81–1.00]**	**510**	**1.00 [1.00–1.00]**	**0.74 [0.57–0.91]**	**1.00 [1.00–1.00]**	**0.67 [0.55–0.86]**	**17 (49)**	**6 (17)**	**0 (0)**	**12 (34)**	**0 [0–0]**
	**Optimal Spe**		**406**	**0.92 [0.75–1.00]**	**0.74 [0.57–0.91]**	**0.95 [0.83–1.00]**	**0.65 [0.50–0.85]**	**17 (49)**	**6 (17)**	**1 (3)**	**11 (31)**	**8 [0–25]**
	Youden		459	1.00 [0.75–1.00]	0.78 [0.61–1.00]	1.00 [0.85–1.00]	0.71 [0.57–1.00]	18 (53)	5 (14)	0 (0)	12 (34)	0 [0–33]
**A.T.**		**0.50 [0.36–0.64]**		**0.18 [0.00–1.00]**	**0.82 [0.00–1.00]**	**0.75 [0.71–0.82]**	**0.25 [0.10–0.60]**	**27 (51)**	**6 (11)**	**9 (17)**	**11 (21)**	**82 [0–100]**

Values are median [interquartile range] and number (percentage). IQR: interquartile range; ROSC: return of spontaneous circulation; SAPS: simplified acute physiology score; ICU: intensive care unit; mRS: modified Rankin Scale; WSLT: withdrawal of life-sustaining treatment. *1 patient with favorable neurological outcome died in the ICU following multiple organ dysfunction syndrome. **All patients with a decision of WLST were not dead during ICU stay.

- At 24 h, NFL had a moderate accuracy, with an AUC of 0.80 [0.68–0.93]. A value of NFL > 1121 pg/mL had a specificity of 1.00 [1.00–1.00] but a low sensitivity of 0.12 [0.02–0.63]. A value of NFL > 250 pg/mL had a high specificity of 0.90 [0.75–0.90] and a higher sensitivity of 0.51 [0.35–0.67].
- At 48 h, NFL had a moderate accuracy, with an AUC of 0.88 [0.77–0.98]. A value of NFL > 1324 pg/mL had a specificity of 1.00 [1.00–1.00] and a sensitivity of 0.56 [0.34–0.78]. A value of NFL > 383 pg/mL had a high specificity of 0.94 [0.81–1.00] with a higher sensitivity of 0.66 [0.50–0.81].
- At 72 h, NFL had a high accuracy, with an AUC of 0.90 [0.81–1.00]. A value of NFL > 510 pg/mL had a specificity of 1.00 [1.00–1.00] and a good sensitivity of 0.74 [0.52–0.91]. A value of NFL > 406 pg/mL had a high specificity of 0.92 [0.75–1.00] and a sensitivity of 0.74 [0.57–0.91].

The prognostic performance of NFL at 48 h (AUC 0.88 [0.77–0.98]) tend to be higher compared to the clinical model including Utstein variables (AUC 0.83 [0.72–0.94]), although this difference did not reach significance (*p* = 0.336, ESM 3 – Figure S4).

### Prognostic accuracy of NSE levels for predicting unfavorable outcome

Performances of NSE values at 24, 48, 72 h in predicting poor outcome are presented in ESM3 - Table S4:

- At 24 h, NSE had a moderate accuracy, with an AUC of 0.79 [0.68–0.90]. A NSE > 50 µg/L had a specificity of 1.00 [1.00–1.00], a sensitivity of 0.44 [0.29–0.61].
- At 48 h, NSE had a moderate accuracy, with an AUC of 0.88 [0.79–0.98]. A NSE > 45 µg/L had a specificity of 1.00 [1.00–1.00] and a sensitivity of 0.68 [0.53–0.82].
- At 72 h, NSE had moderate accuracy, with an AUC of 0.89 [0.76–1.00]. A NSE > 29 µg/L had a specificity of 1.00 [1.00–1.00] and a sensitivity of 0.86 [0.73–1.00].

Prognostic performances of NSE and NFL values at 24, 48, 72 h were not statistically different (ESM3 - Table S5). The prognostic performance of NSE at 48 h (AUC 0.88 [0.79–0.98]) tend to be higher compared to the clinical model including Utstein variables (AUC 0.83 [0.72–0.94]), although this difference did not reach significance (*p* = 0.336, ESM 3 – Figure S4).

### Prognostic accuracy of NFL and NSE levels for predicting favorable outcome

Performances of NFL and NSE levels in predicting good outcome are presented in Table 3a:

**Table 3a:** Prognostic performance of NFL levels in predicting favorable outcome

Time, h	Method	AUC	Cutoff level (pg/mL)	Specificity	Sensitivity	PPV	NPV	Patients, No (%)				
								TP	FN	FP	TN	FNR
**24**	**Youden**	**0.80 [0.68–0.93]**	**82**	**0.75 [0.60–0.95]**	**0.81 [0.60–0.93]**	**0.65 [0.48–0.85]**	**0.79 [0.67–0.89]**	**15 (24)**	**5 (8)**	**8 (13)**	**35 (56)**	**25 [5–40]**
	Se. at 1		16	0.02 [0.12–0.63]	1.00 [1.00–1.00]	0.34 [0.32–0.56]	1.00 [1.00–1.00]	20 (32)	0 (0)	38 (60)	5 (8)	0 [0–0]
**48**	**Youden**	**0.88 [0.77–0.98]**	**307**	**0.88 [0.63–1.00]**	**0.78 [0.59–0.78]**	**0.91 [0.82–1.00]**	**0.67 [0.52–1.00]**	**25 (52)**	**7 (15)**	**2 (4)**	**14 (29)**	**13 [0–38]**
	Se. at 1		42	0.56 [0.34–0.78]	1.00 [1.00–1.00]	0.53 [0.43–0.70]	1.00 [1.00–1.00]	16 (33)	0 (0)	14 (29)	18 (38)	0 [0–0]
**72**	**Youden**	**0.90 [0.81–1.00]**	**459**	**0.83 [0.61–1.00]**	**1.00 [0.75–1.00]**	**0.71 [0.57–1.00]**	**1.00 [0.84–1.00]**	**12 (34)**	**0 (0)**	**4 (11)**	**19 (54)**	**0 [0–25]**
**D.T.**		**0.50 [0.36–0.64]**		**0.82 [0.00–1.00]**	**0.18 [0.00–1.00]**	**0.25 [0.10–0.60]**	**0.75 [0.71–0.82]**	**2 (5)**	**9 (20)**	**6 (14)**	**27 (61)**	**82 [0–100]**

**Table 3b:** Prognostic performance of NSE levels in predicting favorable outcome

Time, h	Method	AUC	Cutoff level (µg/L)	Specificity	Sensitivity	PPV	NPV	Patients, No (%)				
								TP	FN	FP	TN	FNR
**24**	**Youden**	**0.79 [0.68–0.90]**	**39**	**0.68 [0.54–0.85]**	**0.85 [0.65–1.00]**	**0.58 [0.47–0.74]**	**0.91 [0.81–1.00]**	**17 (28)**	**3 (5)**	**13 (21)**	**28 (46)**	**15 [0–35]**
	Lit		17	0.98 [0.93–1.00]	0.10 [0.00–0.25]	0.67 [0.00–1.00]	0.69 [0.67–0.73]	2 (3)	18 (30)	1 (1)	40 (66)	90 [75–100]
**48**	**Youden**	**0.88 [0.79–0.98]**	**28**	**0.85 [0.65–0.97]**	**0.88 [0.69–1.00]**	**0.73 [0.56–0.94]**	**0.94 [0.85–1.00]**	**14 (28)**	**2 (4)**	**5 (5)**	**29 (58)**	**13 [0–31]**
	Lit		17	0.94 [0.85–1.00]	0.19 [0.00–0.38]	0.60 [0.00–1.00]	0.71 [0.67–0.77]	3 (6)	13 (26)	2 (4)	32 (64)	81 [63–100]
	Lit		41	0.71 [0.56–0.85]	0.88 [0.69–1.00]	0.59 [0.46–0.74]	0.93 [0.83–1.00]	14 (28)	2 (4)	10 (20)	24 (48)	13 [0–31]
**72**	**Youden**	**0.89 [0.76–1.00]**	**29**	**0.86 [0.73–1.00]**	**1.00 [1.00–1.00]**	**0.80 [0.67–1.00]**	**1.00 [1.00–1.00]**	**12 (35)**	**0 (0)**	**3 (9)**	**19 (56)**	**0 [0–0]**
			17	0.86 [0.73–1.00]	0.42 [0.17–0.67]	0.63 [0.30–1.00]	0.73 [0.63–0.84]	5 (15)	7 (21)	3 (9)	19 (56)	58 [33–83]
**D.T.**		**0.92 [0.85–0.98]**		**0.83 [0.70–0.97]**	**1.00 [1.00–1.00]**	**0.75 [0.63–0.94]**	**1.00 [1.00–1.00]**	**15 (33)**	**0 ()**	**5 (11)**	**25 (56)**	**0 [0–0–]**

Values are median [interquartile range] and number (percentage). AUC: area under the curve, D.T.: descending trend, TP: true-positive, FN: false-negative, FP: false-positive, FNR: false negative rate, TN: true-negative, PPV: positive predictive value, NPV: negative predictive value, lit.: threshold described in the literature [22, 23]

- NFL levels at 24 and 48 h were both moderately accurate, with an AUC of 0.80 [0.68–0.93] and 0.88 [0.77–0.99], respectively. NFL at 72 h had a high accuracy with an AUC of 0.90 [0.81–1.00]. NFL levels < 82 pg/mL, < 307 pg/mL and < 459 pg/mL at 24, 48 and 72 h were the best compromise between specificity and sensitivity (Table 3a).
- NSE level at 24, 48 and 72 h had a moderate accuracy, with an AUC of 0.79 [0.68–0.90], 0.88 [0.79–0.98] and 0.89 [0.76–1.00], respectively. NSE levels < 39 µg/L, < 28 µg/L and < 29 µg/L at 24, 48 and 72 h were considered as the best compromise between specificity and sensitivity (Table 3b).

### Prognostic accuracy of ascending and descending trends of biomarkers for predicting unfavorable and favorable outcome

An increasing trend in NSE was more accurate than an increasing trend in NFL to predict poor outcome. A decreasing trend in NSE was more accurate than a decreasing trend in NFL levels in predicting good outcome (AUC 0.92 [0.85–0.98] versus 0.50 [0.36–0.64], respectively, *p* < 0.001) (ESM3 - Table S5).

### Relationships between NFL and NSE levels

**Fig. 2:**
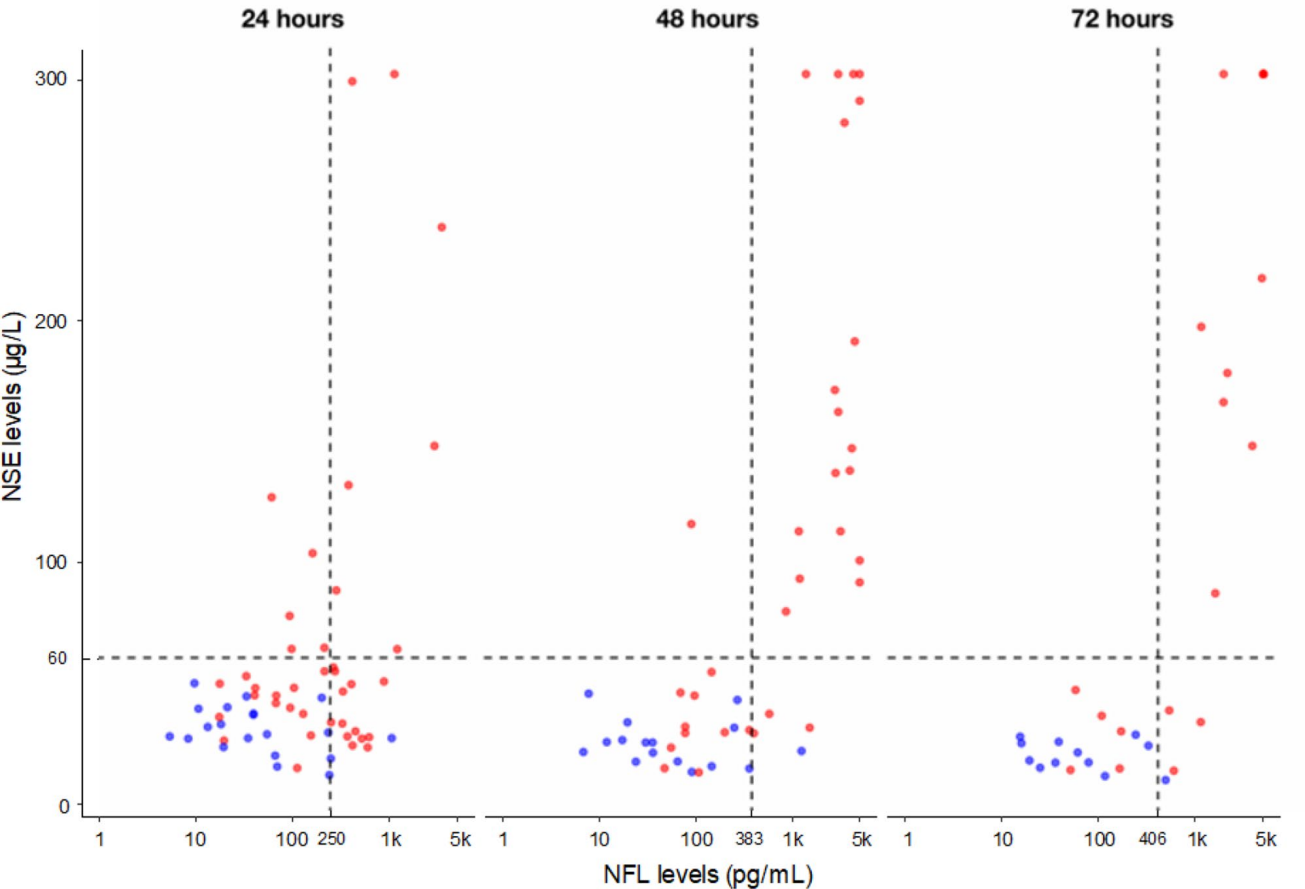
Relationship between NFL and NSE levels at 24, 48, and 72 h after cardiac arrest. Each dot represents a concomitant measurement of NSE and NFL in a same patient. Blue dots represent good outcome (mRS 0–3), while red dots indicate poor outcome (mRS 4–6). To reduce skewness, NFL levels were log₁₀-transformed (1k: 1000 pg/mL; 5k: 5000 pg/mL). Based on 2021 ERC-ESICM [4], we selected a threshold of 60 µg/L for NSE. Based on our results, we determined NFL thresholds with optimal specificity: 250 pg/mL, 383 pg/mL and 406 pg/mL at 24, 48 and 72 h, respectively. Using these thresholds for prediction of poor outcome at each time point (24, 48, and 72 h): *- NSE false* negatives are the red dots located in the lower left and right quadrants; - NFL false negatives are the red dots located in the lower and upper left quadrants; - the false negatives of both NFL and NSE are the red dots located in the lower left quadrant

Relationships and discrepancies between NFL and NSE levels at 24, 48 and 72 h are illustrated in Fig. 2. At 24 h, five patients with a poor outcome presented a high NSE levels (> 60 µg/L) despite low NFL levels (< 250 pg/mL). Conversely, 13 patients with a poor outcome had high NFL levels despite low NSE levels.

### Prognostic accuracy of other prognostic markers

Comparison between the two groups regarding other prognostic markers (i.e., EEG, N20 SSEP, early status myoclonus, corneal and pupillary reflexes) is presented in the ESM3 - Table S6. Compared to patients with a favorable outcome, patients with an unfavorable outcome were more likely to have malignant (68% versus 0%, *p* = 0.001) and highly malignant (12% versus 0%, *p* = 0.001) EEG patterns. Bilateral absence of N20 was more common in patients with an unfavorable outcome (71% versus 0%, *p* = 0.005). We found no significant difference between the two groups regarding bilateral absence of corneal and pupillary reflexes and early status myoclonus. The prognostic performances of these different markers to predict unfavorable outcome are presented in ESM3 - Table S7. A highly malignant EEG pattern predicted an unfavorable outcome with a specificity of 1.00 [1.00–1.00], and a sensitivity of 0.20 [0.08–0.36]. A bilateral abolition of N20 had a specificity of 1.00 [1.00–1.00] and a sensitivity of 0.71 [0.47–0.88]. Finally, early status myoclonus and bilateral corneal and pupillary abolition had a specificity of 0.83 [0.50–1.00] and 0.81 [0.63–1.00] and a sensitivity of 0.30 [0.13–0.52] and 0.26 [0.09–0.43], respectively.

## Discussion

In this prospective cohort of comatose patients after CA, the absolute values of NFL and NSE levels at 24, 48, and 72 h after ROSC were highly predictive of both unfavorable and favorable neurological outcomes, with similar prognostic performances. In addition, the descending and ascending trends of NSE levels over time were predictive of favorable and unfavorable outcomes, respectively. Conversely, NFL kinetics showed limited prognostic value. To our knowledge, this is the first study to assess the prognostic value of both absolute levels and kinetics of these two biomarkers of neuronal injury, for predicting good and poor outcome.

Regarding the prediction of unfavorable outcome, NFL levels at 48 and 72 h after CA demonstrated the highest prognostic value, with an AUC of 0.88 and 0.90, respectively. Thus, NFL levels > 1324 pg/mL at 48 h and > 510 pg/mL at 72 h were 100% specific of an unfavorable outcome, with acceptable sensitivities of 56 and 74%, respectively. These findings are consistent with recent studies reporting AUCs between 0.94 and 0.97 [10–15, 27]. However, these studies identified NFL thresholds values ranging from 970 pg/mL to 1756 pg/mL for equivalent specificity. This discrepancy could be explained by several factors. First, this may be explained by differences in measurement techniques, in particular differences in the immunoassays used for NFL quantification and the signal detection methods applied. In a recent study analyzing blood NFL levels in patients with amyotrophic lateral sclerosis or multiple sclerosis, the method used in this study (Fujirebio reagents on the dedicated Lumipulse G600 II analyzer) demonstrated the strongest correlation with cerebrospinal fluid NFL values. It was also found to be the most sensitive and the least biased of the four tested assays [28]. Second, it could be due to some differences in the population studied. Indeed, this study included IHCA and OHCA patients, whereas the majority of previous studies were *post hoc* analyses of randomized trials that only included OHCA patients [10, 14]. One study compared the prognostic performance of NFL at 48 h between OHCA and IHCA: the prognostic value of NFL to predict poor outcome was lower in IHCA (AUC 0.86) compared to OHCA (AUC 0.97). Thus, it is possible that the prognostic value of NFL is slightly different according to the characteristics of CA. As our study was conducted in an unselected cohort of CA patients, we believe that these results are applicable to the majority of patients who remain comatose at ICU admission after CA. Our results also suggest that NSE levels > 50 µg/L at 24 h, > 45 µg/L at 48 h and > 29 µg/L at 72 h were 100% specific for an unfavorable outcome, with a higher sensitivity compared to the guideline-recommended threshold of 60 µg/L. These results are in line with other studies, suggesting that the threshold of NSE could range from 33 to 120 µg/L [7]. Thus, a personalized approach of NSE according to EEG patterns could improve the prediction of poor outcome [29].

In terms of predicting a favorable outcome, we found that NFL < 82 pg/mL, < 307 pg/mL, and < 459 pg/mL at 24, 48 and 72 h were the best thresholds. These findings are consistent with the study by Moseby-Knappe et al., suggesting that normal NFL levels (< 55 pg/mL) at all time points predicted a favorable outcome, despite a limited sensitivity. For NSE, there is no consensus on optimal cut-off values for predicting favorable outcome. This study suggests that NSE levels < 39 µg/, < 28 µg/L and < 29 µg/L at 24, 48 and 72 h were the most predictive of good outcome, which is consistent with recent studies reporting cut-offs ranging from 17 to 51 µg/L [8, 24, 25]. The identification of biomarkers predictive of favorable outcome is crucial for intensivists, as this information could guide the intensity of care, particularly to avoid inappropriate WLST decisions when biomarkers remain low after CA. Furthermore, the use of these markers predictive of good outcome seems to reduce prognostic uncertainty in comatose patients after CA [8].

Interestingly, we found no significant difference in prognostic performance between absolute levels of NFL and NSE. This finding contrasts with recent studies suggesting a higher prognostic performance of NFL levels compared to NSE. NFL, a neuron-specific structural protein of the cytoskeleton, is released following neuro-axonal damage. In contrast, NSE, an intracellular enzyme, can be derived not only from neurons but also from peripheral neuroendocrine cells, platelets, and erythrocytes. This broader origin makes NSE more susceptible to confounding factors such as hemolysis, particularly in patients receiving extracorporeal membrane oxygenation (ECMO). While some studies have proposed adjusted NSE thresholds for patients on ECMO, caution is warranted when using NSE to predict outcome or guide WLST decisions [30]. No confounding factors (such as hemolysis or ECMO) were identified in this cohort, which may explain why the prognostic performance of these two biomarkers is similar. Other possible explanations include differences in the study populations and methodological biases related to the immunoassays used to measure NFL (described previously), which are important technical limitations when comparing results between studies.

This study also highlighted that the ascending and descending trends of NSE were highly predictive of both poor and good outcomes respectively, with an excellent AUC of 0.92. These results are consistent with some previous studies [8, 16], and highlight the interest of tracking NSE levels during the first three days after CA. Conversely, we found that the dynamic trends in NFL were of limited prognostic value (AUC of 0.50). Data on NFL kinetics after CA remain extremely limited; animal studies suggest that NFL peaks at 48 h post CA [19] but this is not firmly established in humans. Further clinical studies on the NFL kinetics are therefore necessary to confirm our preliminary results. Interestingly, we observed a discrepancy between NFL and NSE levels at 24 h, with some patients showing poor neurological outcomes despite low NSE (< 60 µg/L), but with high NFL (> 250 pg/mL). This may reflect differences in biomarker kinetics and injury localization—NSE indicating neuronal soma damage and NFL reflecting axonal injury - highlighting the value of a multimodal neuroprognostication strategy.

This study has several strengths. First, the prognostic performance of NFL and NSE in predicting a favorable outcome was assessed, as there are few studies on this point [8, 24, 25]. Second, this study described the prognostic value of biomarker kinetics over time, adding some new prognostic information. Third, we compared the prognostic performance of the currently recommended biomarker (i.e., NSE) with that of NFL, a biomarker of great interest for neuroprognostication after CA. Fourth, this prospective study included both OHCA and IHCA patients, whereas previous studies were mainly ancillary studies of randomized trials in OHCA population. Therefore, the thresholds proposed in this study may be relevant for most CA patients. Neurological outcome was assessed by an independent investigator blinded to neuroprognostication data, using the best modified Rankin scale within 3 months. Regarding statistical analysis, the best biomarker threshold was defined by prioritizing the highest specificity (90 and 100%) for predicting poor outcome. Conversely, the Youden index was used to define the best threshold for predicting good outcome prediction, giving the best compromise between specificity and sensitivity. A multimodal approach was used, including markers of structural (i.e., NFL and NSE) and functional (i.e., EEG and SSEP) brain injury [31–33]. Moreover, EEG was assessed by an expert neurophysiologist, according to ACNS terminology and Westhall et al. classification [20, 21]. Finally, common clinical prognostic markers predictive of poor outcome were also collected (i.e., early status myoclonus and bilateral abolition of pupillary and corneal reflexes).

Our study also had some limitations. First, the sample size is limited, and the cohort is monocentric. This may have contributed to the differences in biomarker cut-offs observed in our study compared to those reported in the literature. Second, we chose to include all CA patients who were comatose at ICU admission, rather than only those who remained comatose at 72 h after CA. However, this choice was deliberate because we wanted to assess the prognostic performance of biomarkers as early as 24 h after CA, which required the inclusion of patients at an early stage after admission. Third, we did not evaluate biomarkers of glial injury, such as glial fibrillary acidic protein (GFAP) or S-100 beta protein (S-100 β protein). Nevertheless, previous studies comparing NFL, NSE, GFAP and pS100b suggested that NFL had the highest prognostic value for predicting poor outcome [7, 14, 24]. Fourth, as clinicians were not blinded to NSE values (according to the 2021 ERC-ESICM multimodal neuropronostication algorithm), we cannot exclude the possibility that this may have indirectly improved the prognostic performance of NSE. Finally, we cannot exclude the risk of a self-fulfilling prophecy, although NFL levels were not considered in the neuroprognostication algorithm of this study.

## Conclusion

In this prospective cohort of comatose patients after CA, NFL and NSE levels at 24, 48 and 72 h were highly predictive of favorable and unfavorable neurological outcome. Conversely, only the kinetic of NSE levels between 24 and 72 h after CA was associated with neurological outcome. These results should be confirmed in a larger prospective multicenter study.

## Electronic supplementary material

Below is the link to the electronic supplementary material.

Supplementary Material 1

## Data Availability

Data could be available if request to corresponding author, to a reasonable extent.

## Abbreviations

CA: Cardiac arrest
HIBI: Hypoxic ischemic brain injury
WLST: Withdrawal of life-sustaining treatment
ROSC: Return of spontaneous circulation
SSEP: Somatosensory evoked potentials
NSE: Neuron-specific enolase
NFL: Neurofilament light
EEG: Electroencephalography
GFAP: Glial fibrillary acidic protein
IHCA: In-hospital cardiac arrest
OHCA: Out-hospital cardiac arrest
GCS: Glasgow coma scale
SAPS: Simplified Acute Physiology Score
RASS: Richmond Agitation-Sedation Scale
TTM: Targeted temperature management
ICU: Intensive care unit
ACNS: American Clinical Neurophysiology Society
CPP: Comité de protection des personnes
SE: Status epilepticus
mRS: Modified Rankin scale
ROC: Receiver operating characteristic
AUC: Area under the curve
Se: Sensitivity
Sp: Specificity
PPV: Positive predictive value
NPV: Negative predictive value
FPR: False positive rate
FNR: False negative rate
FP: False positive
FN: False negative
TP: True positive
TN: True negative
PS100b: S100 beta protein
ECLS: Extracorporeal life support
