# Supplementary material for: Absolute blood levels and kinetics of neurofilament light (NFL) chains for neurological prognosis in comatose patients after cardiac arrest
Source: Ann Intensive Care. 2025 May 30;15:75. doi: 10.1186/s13613-025-01491-7 (PMC12125424; doi:10.1186/s13613-025-01491-7)
Supplement: Supplementary file 1 — Supplementary Material 1 [file 13613_2025_1491_MOESM1_ESM.docx]

**Supplementary materials**

ESM1. Management protocol after CA

ESM2. Neurological prognostication algorithm and criteria for WLST

- Decision algorithm for post-resuscitation patients (Figure S1).

ESM3 – Supplementary results

- Figure S2. Flow chart

- Figure S3. Biomarkers levels over time in patients with good (blue line – mRS 0–3) and poor (red line– mRS 4–6) outcomes

- Figure S4. ROC curves for CAHP score, NSE levels at 48h, and NFL levels at 48h, both individually and in combination.

- Table S1. Comparison of demographic characteristics between included and excluded patients.

- Table S2. Biomarkers levels in brain-dead patients at 24, 48, and 72 hours.

- Table S3. Comparison between patients who died following WLST related to supposed severe HIBI and other patients.

- Table S4. Prognostic performances of NSE levels for unfavorable outcome prediction.

- Table S5. Comparison of accuracy of serum biomarkers levels to predict outcome.

- Table S6. Comparison of markers of neuroprognostication between poor and good outcome groups

- Table S7. Prognostic value of usual markers to predict poor outcome.

1. **Management protocol after CA**

Immediately after OHCA and when no obviously other etiology than cardiac origin was retained, all patients had coronary angiography followed by percutaneous coronary intervention if needed. When coronary angiography was inconclusive, brain and angio-thoracic CT scan were performed to identify a reversible cause of CA. TTM was initiated immediately at ICU admission using external cooling by forced cold air during the first 24 hours to obtain a target temperature at 36°C as recommended and subsequently rewarmed to 37°C at a rate of 0.3°C/h. Renal replacement therapy was initiated in case of severe metabolic acidosis and/or in case of life-threatening hyperkalemia, defined as a blood potassium level higher than 6 mmol.L^-1^. A mean arterial blood pressure (MAP) between 65 and 75 mmHg was targeted during the ICU stay. Post-resuscitation shock was defined as a MAP<60 mmHg or a systolic blood pressure<90 mmHg sustained for more than six hours after ROSC, despite adequate fluid loading, and requiring norepinephrine or epinephrine infusion. Convulsive and non-convulsive seizures were treated with antiepileptic drugs (phenytoin, phosphenytoine, levetiracetam, valproate or phenobarbital). During the first 48 hours of ICU stay, treatments were adapted to maintain homeostasis with glucose control, normocapnia, inspired fraction of O_2_ titrated for arterial saturation of 94-98%, mean arterial pressure target to 65-70mmHg, and hemoglobin level over 7 g.dL^-1^.

Concerning sedation regimen, before 2014 we used midazolam and fentanyl with dose titrated to RASS -5 (no response to voice or physical stimulation). For midazolam the infusion starts at 4 mg.h^-1^ and the rate is increased by 1mg.h^-1^ if RASS -4 or by 2mg.h^-1^ if RASS is -3 or more after a bolus of 2 mg. Fentanyl is started at 0,7 μg.kg^-1^.h^-1^ and the titration is made with steps of 25 μg.h^-1^. When the goal is reached we systematically use vecuronium or atracurium to neuromuscular blocking according to train of four responses with 1 or 2 responses. Sedation was interrupted after rewarming and a train of four responses with 4 responses, ensuring that RASS was assessed only after clearance of NMB. In 2014, considering the high incidence of delayed awakening with midazolam-fentanyl, we have decided to change our sedation regimen towards short-acting-drugs. We used after 2014 a sedative protocol with propofol and remifentanil with dose titrated to RASS -5 (no response to voice or physical stimulation). For propofol the infusion starts at 1 mg.kg^-1^.h^-1^ and the rate is increased by 0.1 mg.kg^-1^.h^-1^ with a maximal dose of 4 mg.kg^-1^.h-^1^. Remifentanil is started at 10 μg.kg^-1^.h^-1^ and the titration is made with steps of 1 μg.kg^-1^.h^-1^ with a maximal dose of 18 μg.kg^-1^.h^-1^. When the sedation goal is reached shivering are appraised according to bedside shivering assessment scale (BSAS) every 3hours, with a goal of 0 (no shivering). If goal is not achieved a bolus of Atracurium 0.4 mg.kg^-1^ is given, after two bolus in two hours, an infusion of 0.3 mg.kg^-1^.h^-1^ is started. The titration is made with steps of 0.15 mg.kg^-1^.h^-1^. Sedation was interrupted after rewarming and a train of four responses with 4 responses, ensuring that RASS was assessed only after clearance of NMB.

1. **Neurological prognostication algorithm and criteria for WLST**

After the initial period of TTM and rewarming, neurological outcome is assessed daily in every patient by ICU physicians until death or ICU discharge. At 48 hours after discontinuation of sedation, in patients who do not awake, GCS, pupillary and corneal reflexes are assessed and an SSEP/EEG are performed. We based our neuroprognostication algorithm on post resuscitation care guidelines published on 2021 (see below). An ethic meeting was hold to possibly decide WLST when at least two of the following conditions were observed: no pupillary and corneal reflexes at 72h, bilaterally absent N20 SSEP waves, highly malignant EEG at >24h, status myoclonus ≤ 72h, diffuse and extensive anoxic injury on brain CT/MRI, NSE > 60µg/l at 48h and/or 72h (4). Conversely, when major predictors of poor outcome were not present (i.e., patients with N20 potentials and cranial reflexes preserved, motor GCS more than 2), decisions to withhold or withdraw life-support therapies were systematically delayed in order to search for a confounding factor (sepsis, remaining sedative drug effect, inter-current disease process, other neurological disease). After this additional delay, an ethic meeting was held to incorporate all prognostic variables in the decision. This decision could be either to withhold or withdraw life-support therapies.

WLST was always decided after a collegial decision. All deaths associated with end-of-life decisions occurred during the ICU stay.

**CA with coma**

**Discontinuation of sedation**

**TTM**

**Neurologic examination :**

**- Comatose patients with GCS motor < 3 and a RASS ≤-4**

**2 criteria among:**

**- Bilaterally absent of pupillary and corneal reflexes**

**- NSE level >60µG/L at 48 or/and 72h**

**- Bilaterally absent N20 SSEP waves**

**- highly malignant EEG**

**- diffuse and extensive hypoxic ischemic brain injury**

**- early status myoclonus**

**Withdrawal of life-sustaining treatments**

**Neurologic assesment**

**Continue ICU treatment**

**Reevaluate daily**

**No**

**Yes**

**No**

**Yes**

**Ethic conference**

**Figure S1. Decision algorithm for post-CA patients. Adapted from Nolan et al, Resuscitation and ICM 2021.**

**ESM3 – Supplementary results**

**

**Figure S2. Flow chart**

**A.**

**
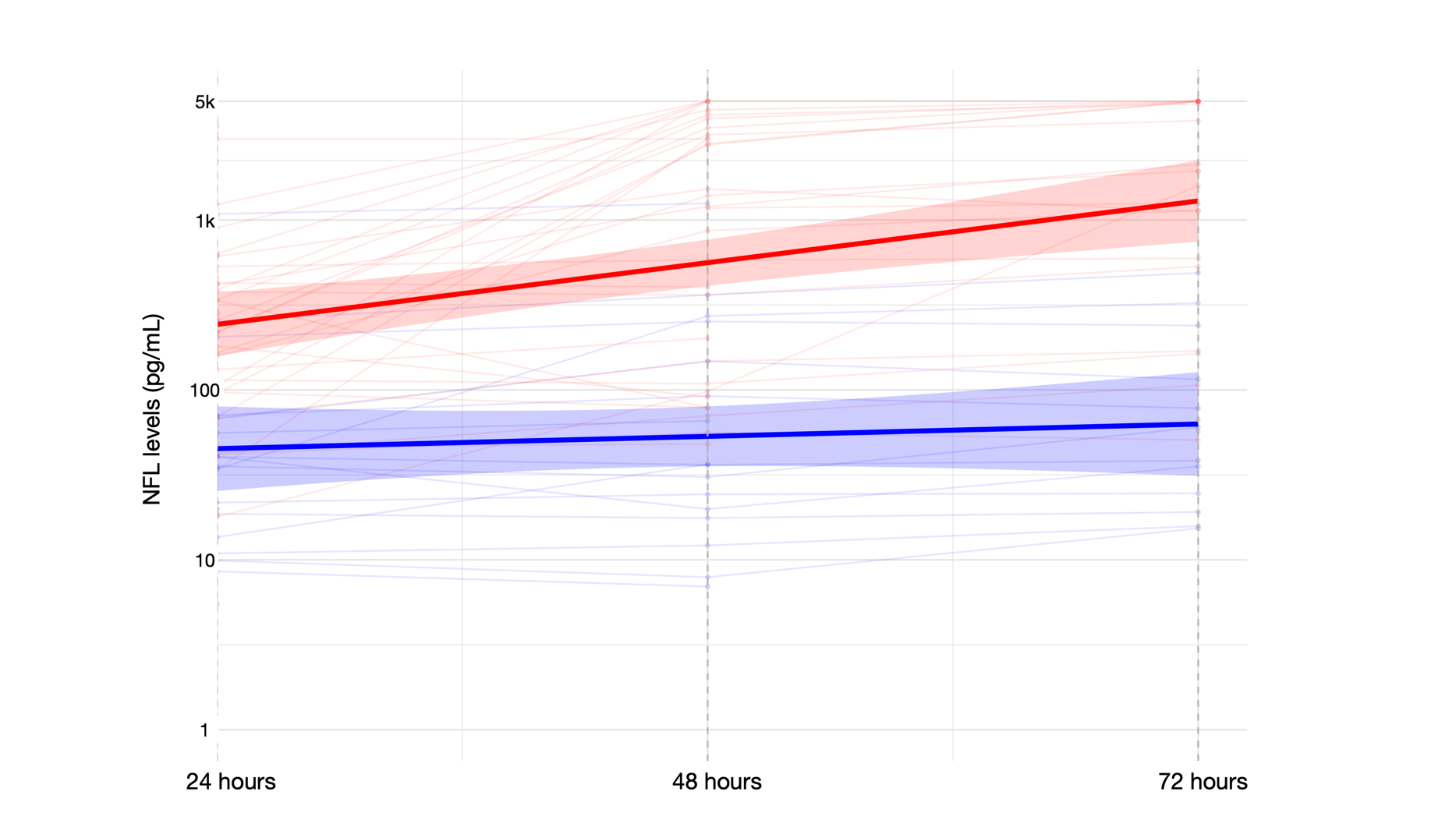
**

**B.**

*
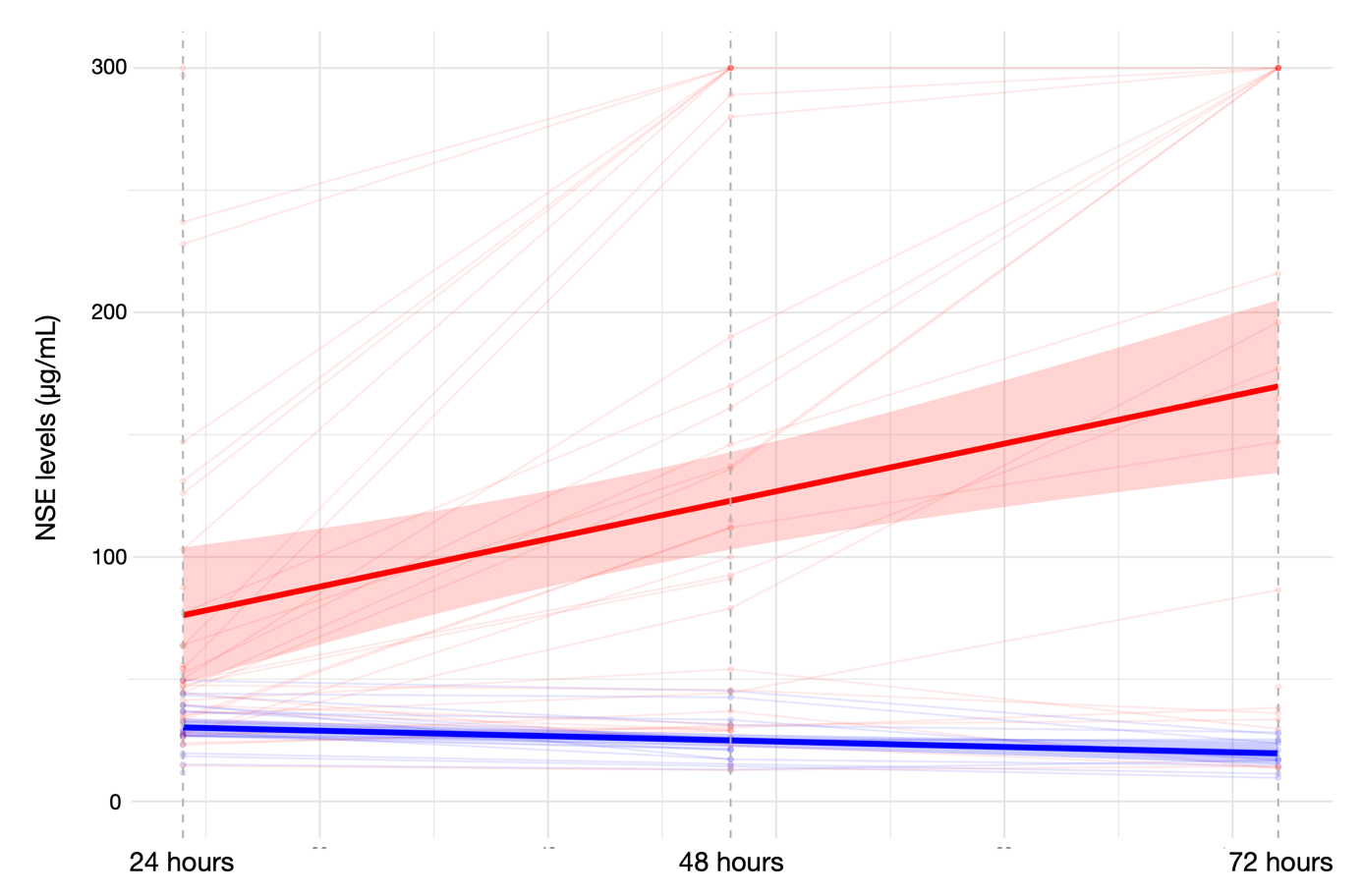
*

**Figure S3. Biomarkers levels over time in patients with good (blue line – mRS 0–3) and poor (red line– mRS 4–6) outcomes.** **A*.*** Neurofilament light (NFL) levels over time. The bold lines represent the trajectory trend with 95% CI. Each dot represents a measurement of biomarker. To reduce skewness, NFL levels were log₁₀-transformed. 1k: 1000 pg/mL; 5k: 5000 pg/mL. **B*.*** Neuron-specific enolase (NSE) levels over time. The bold lines represent the trajectory trend with 95% CI. Each dot represents a measurement of biomarker.

**Figure S4. ROC curves for CAHP score, NSE levels at 48h, and NFL levels at 48h, both individually and in combination. p=0.336.** *NSE: neurone specific enolase; NFL: neurofilament light chain. CAHP : Cardiac Arrest Hospital Prognosis*

|  | Included patients  n=67 | Excluded patients  n=73 | p value |
| --- | --- | --- | --- |
|  |  |  |  |
| Sex – no. (%) |  |  | 1.000 |
| Men | 46 (67) | 49 (67) |  |
| Location at cardiac arrest |  |  | 0.955 |
| Place of residence | 22 (33) | 23 (32) |  |
| Public place | 18 (27) | 23 (32) |  |
| Hospital | 16 (24) | 16 (22) |  |
| Other | 10 (15) | 11 (15) |  |
| Bystander-witnessed cardiac arrest | 62 (94) | 66 (90) | 0.853 |
| Bystander-performed CPR | 54 (82) | 55 (75) | 0.567 |
| **First monitored rhythm – no. (%)** |  |  | **<0.001** |
| Shockable rhythm | 28 (42) | 34 (47) |  |
| Non shockable rhythm | 38 (58) | 39 (53) |  |
| Pulseless electrical activity | 2 (5) | 23 (59) |  |
| Asystole | 36 (95) | 16 (41) |  |
| No flow | 0 [0–5] | 0 [0–3] | 0.942 |
| Low flow | 19 [9–30] | 20 [10–30] | 0.723 |
| Epinephrine – no. (%) | 52 (79) | 47 (64) | 0.346 |
| Arterial pH | 7.23 [7.16–7.34] | 7.22 [7.14–7.34] | 0.262 |
| Arterial lactate level – mmol/liter | 3.7 [1.7–9.6] | 5.5 [2.1–8.9] | 0.076 |
| **Best mRS at 3 months– no. (%)** |  |  | **<0.001** |
| 0 | 13 (20) | – |  |
| 1 | – | 4 (11) |  |
| 2 | 1 (2) | 5 (14) |  |
| 3 | 2 (3) | 9 (24) |  |
| 4 | 10 (15) | – |  |
| 5 | 7 (11) | – |  |
| 6 | 33 (50) | 19 (51) |  |
| MD | 0 (0) | 36 (49) |  |

**Table S1. Comparison of demographic characteristics between included and excluded patients.** *Values are median [interquartile range] and number (percentage). MD: missing data. mRS: modified Rankin Scale; CPR: cardio-pulmonary resuscitation; SAPS II: Simplified Acute Physiology Score.*

|  | Brain death patients |
| --- | --- |
|  | N=9 |
| NSE (IQR) – µg/L |  |
| 24 hours | 107.0 [69.5–166.0] |
| 48 hours | 281.5 [200.2–300.0] |
| 72 hours | 300.0 [300.0–300.0] |
| NFL (IQR) – pg/mL |  |
| 24 hours | 235.0 [43.3–871.0] |
| 48 hours | 849.6 [205.7–4993.0] |
| 72 hours | 4572.0 [3012.0–4786.0] |

**Table S2. Biomarkers levels in brain-dead patients at 24, 48, and 72 hours;** *NSE: neurone specific enolase; NFL: neurofilament light chain*

|  | WLST due to HIBI  n=23 | Other patients  n=44 | p value |
| --- | --- | --- | --- |
|  |  |  |  |
| Age – yr (IQR) | 64 [58–72] | 64 [52–75] | 0.878 |
| Sex – no. (%) |  |  | 0.878 |
| Men | 12 (60) | 33 (70) |  |
| Location at cardiac arrest |  |  | 0.066 |
| Place of residence | 10 (46) | 12 (77) |  |
| Public place | 7 (32) | 11 (5) |  |
| Hospital | 1 (5) | 15 (7) |  |
| Other | 4 (18) | 5 (11) |  |
| Bystander-witnessed cardiac arrest | 19 (87) | 43 (98) | 0.202 |
| Bystander-performed CPR | 15 (68) | 39 (89) | 0.091 |
| First monitored rhythm – no. (%) |  |  | 0.117 |
| Shockable rhythm | 8 (36) | 20 (46) |  |
| Non shockable rhythm | 14 (64) | 24 (54) |  |
| Pulseless electrical activity | 2 (0) | 0 (0) |  |
| Asystole | 12 (0) | 24 (100) |  |
| **Median time from cardiac arrest to sustained ROSC (IQR) – min** | **35 [30–43]** | **19 [8–24]** | **<0.001** |
| **No flow** | **5 [1–10]** | **0 [0–2]** | **<0.001** |
| **Low flow** | **30 [28–35]** | **15 [7–22]** | **<0.001** |
| **Epinephrine – no. (%)** | **22 (100)** | **30 (68)** | **<0.001** |
| SAPS II | 79 [68–87] | 70 [61–82] | 0.058 |
| Arterial pH | 7.20 [7.16–7.26] | 7.28 [7.16–7.36] | 0.181 |
| Arterial lactate level – mmol/liter | 7.7 [3.5–9.5] | 2.7 [1.6–10.0] | 0.060 |
| ICU length of stay (IQR) – days | 4 [3–7] | 4 [3–10] | 0.794 |
| **NSE (IQR) – µg/L** |  |  |  |
| **24 hours** | **51.3 [44.8–96.6]** | **32.6 [26.7–43.8]** | **<0.001** |
| **48 hours** | **153.5 [109.0–291.8]** | **27.5 [21.0–33.0]** | **<0.001** |
| **72 hours** | **300.0 [181.8–300.0]** | **22.4 [14.6–28.7]** | **<0.001** |
| **NFL (IQR) – pg/mL** |  |  |  |
| 24 hours | 218.7 [89.0–469.4] | 100.0 [37.9–312.5] | 0.125 |
| **48 hours** | **3328.7 [1738.8–4416.4]** | **84.9 [36.3–267.3]** | **<0.001** |
| **72 hours** | **4335.5 [1936.0–5000.0]** | **78.0 [38.3–239.3]** | **<0.001** |

**Table S3. Comparison between patients who died following WLST related to supposed severe HIBI and other patients.** *Values are median [interquartile range] and number (percentage). WLST: withdrawal of life sustaining therapies; NSE: neurone specific enolase; NFL: neurofilament light chain; ICU: intensive care unit; CPR: cardio-pulmonary resuscitation; SAPS II: Simplified Acute Physiology Score.*

| **Time, h** | Method | AUC | Cutoff level | Specificity | Sensitivity | PPV | NPV | Patients, No (%) | | | | |
| --- | --- | --- | --- | --- | --- | --- | --- | --- | --- | --- | --- | --- |
|  |  | | | | |  |  | TP | FN | FP | TN | FPR |
| **24** | Spe. at 1 | 0.79 [0.68–0.90] | 50 | 1.00 [1.00–1.00] | 0.44 [0.29–0.59] | 1.00 [1.00–1.00] | 0.46 [0.41–0.54] | 18 (29) | 23 (38) | 0 (0) | 20 (33) | 0 [0–0] |
|  | Optimal Spe |  | 44 | 0.90 [0.75–0.90] | 0.61 [0.46–0.76] | 0.93 [0.83–1.00] | 0.53 [0.44–0.65] | 25 (41) | 16 (26) | 2 (3) | 18 (30) | 10 [0–25] |
|  | Youden |  | 39 | 0.85 [0.65–1.00] | 0.71 [0.54–0.85] | 0.91 [0.80–1.00] | 0.58 [0.47–0.73] | 29 (48) | 12 (20) | 3 (5) | 17 (28) | 15 [0–35] |
| **48** | Spe. at 1 | 0.88 [0.79–0.98] | 45 | 1.00 [1.00–1.00] | 0.68 [0.53–0.82] | 1.00 [1.00–1.00] | 0.59 [0.50–0.73] | 23 (46) | 11 (22) | 0 (0) | 16 (32) | 0 [0–0– |
|  | Optimal Spe |  | 44 | 1.00 [1.00–1.00] | 0.68 [0.53–0.82] | 1.00 [1.00–1.00] | 0.59 [0.50–0.73] | 24 (48) | 10 (20) | 1 (2) | 15 (30) | 6 [0–19] |
|  | Youden |  | 28 | 0.88 [0.69–1.00] | 0.85 [0.65–0.97] | 0.94 [0.86–1.00] | 0.74 [0.56–0.93] | 29 (58) | 5 (10) | 2 (4) | 14 (28) | 13 [0–31] |
|  | Litt |  | 60 | 1.00 [1.00–1.00] | 0.62 [0.47–0.79] | 1.00 [1.00–1.00] | 0.55 [0.47–0.70] | 21 (42) | 13 (26) | 0 (0) | 16 (32) | 0 [0–0] |
| **72** | Spe. at 1 | 0.89 [0.76–1.00] | 29 | 1.00 [1.00–1.00] | 0.86 [0.73–1.00] | 1.00 [1.00–1.00] | 0.80 [0.67–1.00] | 19 (56) | 3 (9) | 0 () | 12 (35) | 0 [0–0] |
|  | Optimal Spe |  | 28 | 0.92 [0.75–1.00] | 0.86 [0.68–1.00] | 0.95 [0.86–1.00] | 0.79 [0.61–1.00] | 19 (56) | 3 (9) | 1 (3) | 11 (32) | 8 [0–25] |
|  | Youden |  | 29 | 1.00 [1.00–1.00] | 0.86 [0.73–1.00] | 1.00 [1.00–1.00] | 0.80 [0.67–1.00] | 18 (55) | 3 (9) | 0 (0) | 12 (36) | 0 [0–0] |
|  | Litt |  | 60 | 1.00 [1.00–1.00] | 0.64 [0.45–0.81] | 1.00 [1.00–1.00] | 0.60 [0.50–0.75] | 14 (42) | 8 (24) | 0 (0) | 12 (35) | 0 [0–0] |
| **A.T.** |  | 0.92 [0.85–0.98] |  | 1.00 [1.00–1.00] | 0.83 [0.70–0.97] | 1.00 [1.00–1.00] | 0.75 [0.63–0.94] | 25 (56) | 5 (11) | 0 (0) | 15 (33) | 0 [0–0] |

**Table S4. Prognostic performances of NSE levels for unfavorable outcome prediction.** *Values are median [interquartile range] and number (percentage). AUC: area under the curve, Lit.: literature, A.T.: ascending trend, TP: true-positive, FN: false-negative, FP: false-positive, TN: true-negative, PPV: positive predictive value, NPV: negative predictive value, spe: specificity, litt: threshold according to literature.*

|  | NSE | NFL | p-value |
| --- | --- | --- | --- |
| AUC at 24 h | 0.79 [0.68–0.90] | 0.80 [0.68–0.93] | 0.889 |
| AUC at 48 h | 0.88 [0.79–0.98] | 0.88 [0.77–0.98] | 0.978 |
| AUC at 72 h | 0.89 [0.76–1.00] | 0.90 [0.81–1.00] | 0.662 |
| Trend | 0.92 [0.85–0.98] | 0.50 [0.36–0.64] | <0.001 |

**Table S5. Comparison of accuracy of serum biomarkers levels to predict outcome.** *Values are median [interquartile range]. AUC: area under the curve; NSE: neuron-specific enolase; NFL: neurofilament light chain.*

|  | All patients | Favorable | Unfavorable | p-value |
| --- | --- | --- | --- | --- |
|  | N=67 | N=16 | N=51 |  |
| **NSE (IQR) – ng/mL** |  |  |  |  |
| **24 hours** | **39.4 [27.6–54.4]** | **28.9 [25.8–37.5]** | **47.6 [33.4–64.1]** | **<0.001** |
| **48 hours** | **43.5 [25.2–136.8]** | **23.4 [17.3–27.4]** | **106.0 [32.9–185.0]** | **<0.001** |
| **72 hours** | **34.8 [18.5–211.0]** | **19.4 [16.3–25.0]** | **171.0 [36.7–300.0]** | **<0.001** |
| **NFL (IQR) – pg/mL** |  |  |  |  |
| **24 hours** | **158.5 [41.0–382.7]** | **37.9 [17.4–104.5]** | **256.0 [96.2–441.9]** | **<0.001** |
| **48 hours** | **317.2 [69.2–2862.0]** | **36.4 [19.3–174.0]** | **1297.7 [137.6–3605.0]** | **<0.001** |
| **72 hours** | **487.7 [63.7–2032.5]** | **49.3 [23.2–146.4]** | **1591.9 [350.6–4913.5]** | **<0.001** |
| **EEG performed – no. (%)** | **29 (44)** | **4 (20)** | **25 (54)** | **0.021** |
| Time to first EEG (IQR) – days | 1 [1–3] | 2 [1–2] | 1 [1–3] | 0.944 |
| Continuity – no. (%) |  |  |  | 0.145 |
| Continuous | 11 (41) | 4 (100) | 7 (30) |  |
| Nearly continuous | 2 (7) | 0 (0) | 2 (9) |  |
| Discontinuous | 10 (37) | 0 (0) | 10 (44) |  |
| Suppression-burst | 2 (7) | 0 (0) | 2 (9) |  |
| Suppression | 2 (7) | 0 (0) | 2 (9) |  |
| **Voltage – no. (%)** |  |  |  | **0.018** |
| Normal | 10 (37) | 4 (100) | 6 (26) |  |
| Low | 12 (44) | 0 (0) | 12 (52) |  |
| Suppressed | 5 (19) | 0 (0) | 5 (33) |  |
| Isoelectric | 0 (0) | 0 (0) | 0 (0) |  |
| Rhythmic and periodic discharges – no. (%) | 8 (29) | 0 (0) | 8 (33) | 0.442 |
|  |  |  |  |  |
| EEG reactivity – no. (%) | 17 (65) | 4 (100) | 13 (59) | 0.312 |
| **Westhall classification – no. (%)** |  |  |  | **0.001** |
| **Benign** | **5 (17)** | **4 (100)** | **3 (12)** |  |
| **Malignant** | **17 (59)** | **0 (0)** | **17 (68)** |  |
| **Highly malignant** | **7 (24)** | **0 (0)** | **3 (12)** |  |
| Bilateral absence of corneal reflex – no. (%) | 9 (13) | 3 (18) | 6 (13) | 0.882 |
| Early status myoclonus – no. (%) | 8 (28) | 1 (25) | 7 (28) | 0.519 |
| Bilateral absence of pupillary light reflex – no. (%) | 11 (16) | 4 (24) | 7 (15) | 0.993 |
| Time to SSEP (IQR) – days | 3 [3–4] | 2 [2–5] | 3 [3–4] | 0.485 |
| **SSEP – no. (%)** | **20 (30)** | **3 (15)** | **17 (37)** | **0.005** |
| **Bilateral absence of N20** | **12 (60)** | **0 (0)** | **12 (71)** |  |
| **Bilateral presence of N20** | **5 (25)** | **0 (0)** | **3 (18)** |  |

**Table S6. Comparison of markers of neuroprognostication between poor and good outcome groups.** *Values are median [interquartile range] and number (percentage). 
NSE: neuron-specific enolase; NFL: neurofilament light chain; IQR: interquartile range; EEG: electroencaphlogram; SSEP: somatosensory evocated potential.*

|  | AUC | Specificity | Sensitivity | NPV | PPV | FPR |
| --- | --- | --- | --- | --- | --- | --- |
| Highly malignant EEG | 0.60 [0.52–0.68] | 1.00 [1.00–1.00] | 0.20 [0.04–0.36] | 0.17 [0.14–0.20] | 1.00 [1.00–1.00] | 0 [0–0] |
| No pupillary and corneal reflexes | 0.54 [0.40–0.67] | 0.81 [0.63–1.00] | 0.26 [0.09–0.43] | 0.43 [0.34–0.52] | 0.67 [0.38–1.00] | 19 [0–38] |
| Bilateral absent N20 SSEP | 0.85 [0.74–0.96] | 1.00 [1.00–1.00] | 0.71 [0.47–0.94] | 0.38 [0.25–0.75] | 1.00 [1.00–1.00] | 0 [0–0] |
| Early status myoclonus | 0.56 [0.38–0.76] | 0.83 [0.50–1.00] | 0.30 [0.13–0.52] | 0.24 [0.16–0.33] | 0.89 [0.67–1.00] | 17 [0–50] |

**Table S7. Prognostic value of usual markers to predict poor outcome.** *Values are median [interquartile range]. EEG: electroencephalogram, SSEP: somatosensory evoked potential; AUC: area under the curve; NPV: negative predictive value; PPV: positive predictive value; FPR: false-positive rate.*
